# The UK National Mouse Genetics Network: Translating genetic findings using preclinical mouse models to better understand neurodevelopmental, neuropsychiatric and neurodegenerative disorders

**DOI:** 10.1177/23982128251383973

**Published:** 2025-11-30

**Authors:** Sevda T. Boyanova, Nicholas E. Clifton, Karolina Dec, Michal M. Milczarek, MURIDAE Cluster, Anthony R. Isles

**Affiliations:** 1 UK Dementia Research Institute at University College London, London, UK; 2 UCL Queens Square Institute of Neurology, University College London, London, UK; 3 Faculty of Health and Life Sciences, University of Exeter, Exeter, UK; 4 Neuroscience and Mental Health Innovation Institute, School of Medicine, Cardiff University, Cardiff, UK; 5 School of Physiology, Pharmacology and Neuroscience, Faculty of Health and Life Sciences, University of Bristol, Bristol, UK

The past two decades have seen an explosion of genomic data providing insight into the biological mechanisms that underpin human disease (Claussnitzer et al., 2020; Kamatani and Okada, 2021). To exploit this valuable resource, preclinical animal models are an essential tool to understand disease mechanisms and develop novel therapies. Here, genetic mouse models play a vital role, sharing a highly similar genomic architecture to humans while providing an intact physiological system in which to ask questions regarding the impact of genetic variants linked to disease. The National Mouse Genetics Network (NMGN) was conceived as a new funding model for mouse genetics by the Medical Research Council (MRC), that would be able to exploit the UK’s world-leading scientific and clinical expertise to investigate defined disease areas and improve and accelerate how research using animals is translated to clinical benefits (Sansom et al., 2024). The NMGN aims to create a broad platform for open access to mouse genetics for translational medicine and is made of a set of research clusters, including some of direct relevance to the neuroscience community. The Mary Lyon Centre (MLC) at MRC Harwell acts as a central hub for the network as well as being a repository for mouse resources with an established international reach. The MLC generates novel mouse strains closely reflective of human diseases and develops complex phenotyping platforms that can be used by the different clusters, while facilitating high-quality data collection and sharing.

In partnership with the BNA (British Neuroscience Association), the MURIDAE (Modalities for Understanding, Recording and Integrating Data Across Early life) cluster organised a symposium at the recent International BNA2025 Festival of Neuroscience (BNA2025) in Liverpool to showcase the neuroscience-focused research of the NMGN and highlight opportunities by which the neuroscience community could collaborate and interact with the Network. The four speakers, who were all early career researchers, represented some of the different possible levels of interaction with the NGMN.

MURIDAE post-doc, Dr Michal Milczarek, currently based at the University of Bristol, gave an overview of his exciting work developing home-cage monitoring of young, pre-weaning mice. Creating a pipeline for the analysis of behavioural development using home-cage analysis is a central objective of the MURIDAE cluster and builds on the expertise of the MLC (Hobson et al., 2020). In particular, Dr Milczarek and other MURIDAE members will be applying these tools to novel genetic mouse models for schizophrenia also generated by the MLC. Dr Karolina Dec, a Cardiff University post-doc funded by the Business Engagement Fund which is aimed at forging links between the NMGN and industry, talked about her project developing human induced pluripotent stem cell (hiPSC)-derived neuronal models that carry the same genetic variants as the MURIDAE mouse models. By testing the synaptic function and network interactions in the hiPSC-derived cortical neurons using multi-electrode arrays (Dec et al., 2023), the aim is to streamline drug development before moving to *in vivo* systems.

The UK Dementia Research Institute (UK DRI) is an associate cluster of the NGMN and has additional links via the Ageing cluster. Dr Sevda Boyanova, a Research Fellow with Dr Frances Wiseman at UCL, gave a detailed overview of the behavioural profiling of mouse models being undertaken by the UK DRI (Boyanova et al., 2025). This comprehensive pipeline of tests, ranging from one-off tests of spontaneous behaviour to cognitive measures using touchscreen-based tasks, was coordinated and carried out at the MLC; this systematic approach is being applied across multiple mouse models. These data will facilitate mapping of mouse models to disease mechanisms and developmental stage, aiding preclinical research for the early treatment of dementia-causing diseases.

In addition to the core activity of the constitutive clusters, a central tenet of the NMGN mission is to be a touchstone for preclinical researchers across the UK and further afield. This will be achieved by open sharing of data, resources, and access to courses to accelerate advances and train and retain world-class skills in mouse genetics. The main route for researchers to link with the NMGN is to become associate members (MRC National Mouse Genetics Network, 2022). The last speaker at our symposium, Dr Nicholas Clifton, is one such member who has combined analyses of human and mouse model “omics” data. This approach has previously yielded interesting insights into the aetiology of schizophrenia (Clifton et al., 2024, 2025), and work is ongoing to apply these techniques to the mouse models generated as part of the MURIDAE cluster.

At BNA2025, the strength and importance of using preclinical models in neuroscience was prominent; in addition to our symposium, a session was hosted by The Wellcome Trust, who recently commissioned a report into how to best harness the potential from *in silico*, cellular and animal models (Gu et al., 2023). Our aim was to illustrate the central role the NMGN can play in the development and phenotyping of genetic mouse models, and the variety of ways the neuroscience community can become involved.
